# A comparative histochemical evaluation of tissue biomarkers in normal and pathological specimens: A retrospective study

**DOI:** 10.6026/973206300223072

**Published:** 2026-05-31

**Authors:** Amit Kumar Srivastava, Aishwarya Srivastava, Rohini Srivastava, Prabha Verma, Jyoti Batra, Yogesh Yadav

**Affiliations:** 1 Department of Anatomy, Government Medical College, Datia, Madhya Pradesh, India; 2 Department of Biochemistry, Naraina Medical College and Research Centre, Kanpur, Uttar Pradesh, India; 3 Department of Pathology, Naraina Medical College and Research Centre, Kanpur, Uttar Pradesh, India; 4 Department of Biochemistry, GSVM Medical College, Kanpur, Uttar Pradesh, India; 5 Department of Central Research Facility, Santosh Deemed to be University, Ghaziabad, Uttar Pradesh, India; 6 Department of Anatomy, Saraswathi Institute of Medical Sciences, Hapur, Uttar Pradesh, India

**Keywords:** Histochemistry, tissue biomarkers, pathological specimens, normal tissue, retrospective study

## Abstract

Differences in histochemical biomarker expression between normal and pathological tissues remain inadequately characterized. Therefore, it is of interest to evaluate histochemical expression patterns of selected biomarkers in normal and pathological tissue samples. Hence, eighty archived specimens (40 normal and 40 pathological) were analyzed using histochemical staining and semi-quantitative scoring over two years. Pathological tissues showed higher staining intensity and altered distribution patterns, indicating changes in cellular metabolism and extracellular matrix composition. Thus, we show distinct biomarker expression patterns in diseased tissues, supporting their diagnostic and prognostic relevance.

## Background:

Histochemical analysis is a valuable diagnostic and research method in pathology to investigate the chemical composition and distribution of biomolecules in tissues. It enables the visualization of certain cellular components including proteins, lipids, carbohydrates and enzymes within the tissues sections with the help of special staining techniques. These techniques have been extensively employed to comprehend the physiological and pathological changes in tissues that happen in different diseases [1]. Tissue biomarkers refer to biological molecules that reflect normal or abnormal biological functions, disease conditions or reaction to therapeutic measurements. They are critical in the diagnosis, prognosis and follow-up of the treatment outcomes of disease. Histochemical methods can be used to localize these biomarkers in tissues and are valuable in giving information on cellular and extracellular alterations in disease conditions [2]. Cellular structures and biochemical components in normal tissues are organized to show well-organized distribution patterns, which are indicators of physiological homeostasis. But in sickness, tissue architecture and biochemical composition are frequently changed due to pathological processes that involve inflammation, degeneration and neoplasia. Such alterations are observable by use of histochemical staining where differences in biomarker expression in normal and disease tissues are observed [3]. Histochemical methods have played a very important role in the study of tissue pathology over the last few decades.

Periodic acid-Schiff (PAS), Masson's trichrome staining and enzyme histochemistry have been extensively employed to assess extracellular matrix contents, connective tissue fibers and cellular metabolic activity in normal and pathological tissues [4]. They are used to give knowledge on structural alterations that occur in tissues and are used to determine the pathological processes at a microscopic level. The comparative analysis of tissue biomarkers in normal and pathological specimens is especially important in the study of the pathogenesis of diseases. Various studies have shown that changes in the expression of biomarkers can be indicative of disease progression, tissue damage and regeneration [5]. The detection of this variation may help differentiate between normal physiological and pathological alteration. Histochemical and immunohistochemical studies have become more and more significant in biomedical research in recent years. Such methods enable a comprehensive description of molecular and cellular changes in tissues and lead to a better diagnosis in pathology. Comparative histochemical analysis also assists in setting baseline patterns of biomarker expression in normal tissues, which are critical in elucidating pathological alterations [6]. Even with the development of molecular diagnostics, histochemical methods are commonly employed because of their simplicity, low cost and the possibility of the direct visualization of tissue components. Therefore, evaluating differences in biomarker expression between normal and pathological tissues continues to be an important area of research in pathology and biomedical sciences [7]. Therefore, it is of interest to conduct a comparative histochemical analysis of tissue biomarkers in normal and pathological samples to establish variations in staining patterns and expression intensities that can be used to enhance our knowledge on disease mechanism.

## Materials and Methodology:

## Study design:

The present study was designed as a retrospective comparative histochemical study conducted to evaluate and compare the expression of selected tissue biomarkers in normal and pathological tissue specimens.

## Study setting:

The study was carried out in the Department of Pathology/Histopathology of a tertiary care teaching hospital. Archived tissue specimens preserved in the departmental histopathology records were used for analysis.

## Study duration:

The study was conducted over a period of two years, during which archived tissue specimens collected in the previous years were retrieved and analyzed.

## Sample size:

A total of 80 tissue specimens were included in the study. These consisted of:

[1] 40 normal tissue specimens (control group)

[2] 40 pathological tissue specimens (study group)

The sample size was determined based on previously published histochemical studies comparing biomarker expression in normal and diseased tissues and considering feasibility and availability of archived specimens.

## Sampling technique:

Archived formalin-fixed paraffin-embedded (FFPE) tissue blocks were retrieved from the histopathology archives using purposive sampling based on inclusion and exclusion criteria.

## Inclusion criteria:

[1] Adequately preserved formalin-fixed paraffin-embedded tissue blocks.

[2] Tissue specimens with confirmed histopathological diagnosis.

[3] Normal tissue specimens obtained from non-diseased surgical margins or normal biopsies.

[4] Pathological tissue specimens showing clear histopathological changes.

## Exclusion criteria:

[1] Poorly preserved tissue blocks or damaged sections.

[2] Tissue samples with inadequate cellularity.

[3] Specimens showing extensive necrosis or tissue autolysis.

[4] Previously processed tissues with incomplete staining quality.

## Tissue processing and sectioning:

The selected formalin-fixed paraffin-embedded tissue blocks were sectioned using a rotary microtome to obtain 4-5 µm thick sections. The sections were mounted on glass slides coated with adhesive and dried at appropriate temperatures to ensure proper fixation to the slide surface.

## Histochemical staining procedure:

The prepared tissue sections were subjected to routine histochemical staining techniques to evaluate tissue biomarkers and structural components. The staining procedures included:

[1] Hematoxylin and Eosin (H&E) staining for evaluation of general tissue architecture.

[2] Periodic Acid-Schiff (PAS) staining to detect glycogen and carbohydrate-rich biomolecules.

[3] Masson's trichrome staining for assessment of connective tissue and collagen fibers.

The staining procedures were performed according to standard laboratory protocols to ensure uniformity and reproducibility.

## Microscopic evaluation:

The stained slides were examined under a light microscope by experienced pathologists. The evaluation included assessment of:

[1] Staining intensity

[2] Distribution of biomarker expression

[3] Cellular and extracellular structural changes 

## The staining intensity was graded using a semi-quantitative scoring system as follows:

[1] 0 - No staining

[2] 1 - Mild staining

[3] 2 - Moderate staining

[4] 3 - Strong staining 

## Data collection:

Data regarding tissue type, staining intensity and histochemical characteristics were recorded in a standardized data collection sheet. Observations from normal and pathological tissues were compared for differences in biomarker expression patterns.

## Statistical analysis:

All collected data were entered into a Microsoft Excel spreadsheet and analyzed using Statistical Package for the Social Sciences (SPSS) software version 26.0 (IBM Corp., Armonk, NY, USA). Descriptive statistics such as mean, standard deviation and percentages were calculated. Comparative analysis between normal and pathological groups was performed using appropriate statistical tests such as the Chi-square test or Student's t-test, depending on the type of data. A p-value < 0.05 was considered statistically significant.

## Results:

A total of 80 tissue specimens were analyzed in this retrospective study, including 40 normal tissue samples and 40 pathological tissue samples. Histochemical evaluation was performed using Hematoxylin & Eosin (H&E), Periodic Acid-Schiff (PAS) and Masson's trichrome staining techniques to assess the expression of tissue biomarkers and structural components. Special stains such as PAS are useful for detecting glycogen and mucopolysaccharides in tissues, producing a characteristic magenta coloration when these molecules are present Table 1 shows that the study population consisted of an equal distribution of normal and pathological tissue specimens. Equal representation ensured unbiased comparative evaluation of histochemical biomarker expression between both groups. This distribution allowed a balanced statistical analysis of staining intensity and biomarker expression. PAS staining intensity differed significantly between normal and pathological tissues. Strong PAS positivity was observed in 30% of pathological tissues compared to only 7.5% of normal tissues. This indicates increased carbohydrate and mucopolysaccharide accumulation in diseased tissues, which may reflect metabolic alterations or extracellular matrix remodeling associated with pathological processes. Statistical analysis demonstrated a significant difference (p = 0.002) between the groups (Table 2). Masson's trichrome staining demonstrated significantly higher collagen deposition in pathological tissues compared with normal tissues. Approximately 42.5% of pathological samples showed extensive collagen deposition, whereas only 15% of normal tissues exhibited this pattern. This suggests that fibrosis and extracellular matrix remodeling are important features of pathological tissue changes. The difference between groups was statistically significant (p = 0.001) (Table 3).

## Discussion:

Histochemical analysis is significant in the detection of structural and biochemical changes that take place in diseased tissues. The current retrospective study involved the comparison of histochemical expression of the tissue biomarkers under normal and pathological tissue specimens through routine and special staining techniques. In the current research, staining intensity of PAS was found to be significantly more in the pathological tissues than in normal tissues. Strong PAS positivity was found in approximately 30% of pathological tissues which resulted in more carbohydrate and mucopolysaccharide buildup. PAS is a popular technique in histopathology to recognize glycogen, mucins and glycoproteins in tissues and is important in identifying pathological changes in epithelial and connective tissue [8, 9]. In a few of the studies, similar observation was made in which mucin expression of colorectal carcinoma was assessed using PAS staining and revealed a significant increase in PAS positivity of the cancerous tissues over normal mucosa. The authors indicated that abnormal accumulation of mucopolysaccharides could be a manifestation of metabolic and structural changes that occur with tumor progression [10, 11]. Increased collagen deposition in pathological tissues was also exhibited by the results of the current study through trichrome staining of Masson. The proportion of pathological specimens with extensive collagen deposition was about 42.5% versus 15% with normal tissues. Masson trichrome stain has been extensively used to assess connective tissue fibers and fibrosis in different pathological conditions. These results are similar to the research by Chang *et al.*(2008) [2], who have shown that Masson trichrome staining may be used to distinguish fibrous tissue elements of pathological lesions of the head and neck area (PubMed). Their study highlighted the importance of special stains in identifying collagen deposition and distinguishing between different types of soft tissue lesions. The other study by Cohen (2005) showed that the Masson trichrome staining is a useful method to detect structural changes and fibrosis of renal biopsy samples. According to the author, this stain can be useful in terms of connective tissue remodeling and pathological alterations in tissue structure [4]. Likewise, Mao *et al.* assessed the distribution of collagen by trichrome staining of Masson in intestinal diseases and have indicated that collagen fiber patterns are greatly different in inflammatory and normal tissues. The authors concluded that special staining techniques can provide important diagnostic information in differentiating pathological conditions [3]. The current research also noted that there was a substantial variation in the histochemical staining patterns of normal and pathological tissues as evaluated by the routine H&E staining. H&E staining is still the gold standard method in histopathology to assess the tissue architecture and cellular morphology. Other special stains like PAS and trichrome stains give additional diagnostic information by emphasizing on biochemical and structural elements in tissues. The significance of using both histochemical and immunohistochemical methods in enhancing diagnostic accuracy has been highlighted in recent histopathological studies. As an example, a comparative study of staining methods has shown that although standard stains can provide morphological information, special stains can provide more biochemical information that enhances the diagnostic interpretation [12, 13]. The results of the current study substantiate the earlier studies that indicate that histochemical staining techniques are still useful in pathology laboratories. The methods are comparatively cheap, simple to carry out and offer helpful data on tissue structure, disease pathophysiology and changes in structure. The findings also emphasize the role of comparative analysis of normal tissues and pathological tissues in the mechanism of diseases. The presence of higher deposition of mucopolysaccharide and collagen in pathological tissues can be adaptive mechanisms to tissue damage or inflammation [14, 15]. On the whole, the current analysis has shown that histochemical analysis of tissue biomarkers can be an invaluable source of information about structural and biochemical alterations in diseased tissues. These analyses may also help increase the quality of diagnostic tests and enhance knowledge of pathogenesis of diseases.

## Conclusion:

We show significantly higher PAS staining in pathological tissues, indicating increased carbohydrate and mucopolysaccharide accumulation, along with stronger Masson's trichrome staining consistent with collagen deposition, fibrosis and extracellular matrix remodeling (p < 0.05). These results highlight the diagnostic value of histochemical techniques for detecting structural and biochemical alterations in diseased tissues and for improving pathological interpretation. Further large-scale studies employing advanced immunohistochemical markers are warranted for the understanding of tissue biomarker dynamics and enhance diagnostic precision across diverse pathological conditions.

## Figures and Tables

**Table 1:** Distribution of tissue specimens

**Tissue Category**	**Number of samples**	**Percentage**
Normal tissues	40	50%
Pathological tissues	40	50%
Total	80	100%

**Table 2:** Comparison of PAS staining intensity in normal and pathological tissues

**PAS Staining Intensity**	**Normal tissue (n=40)**	**Pathological tissue (n=40)**	**Percentage difference**	**p value**
Negative (Score 0)	12 (30%)	3 (7.5%)	22.50%	
Mild (Score 1)	15 (37.5%)	8 (20%)	17.50%	
Moderate (Score 2)	10 (25%)	17 (42.5%)	17.50%	
Strong (Score 3)	3 (7.5%)	12 (30%)	22.50%	0.002

**Table 3:** Comparison of masson's trichrome staining (Collagen deposition)

**Collagen Deposition**	**Normal tissue (n=40)**	**Pathological tissue (n=40)**	**Percentage difference**	**p value**
Minimal	18 (45%)	6 (15%)	30%	
Moderate	16 (40%)	17 (42.5%)	2.50%	
Extensive	6 (15%)	17 (42.5%)	27.50%	0.001
